# Supplementary material for: Psycho-Educational and Rehabilitative Intervention to Manage Cancer Cachexia (PRICC) for Advanced Patients and Their Caregivers: Lessons Learned from a Single-Arm Feasibility Trial
Source: Cancers (Basel). 2023 Mar 30;15(7):2063. doi: 10.3390/cancers15072063 (PMC10093308; doi:10.3390/cancers15072063)
Supplement: Supplementary file 1 [file cancers-15-02063-s001.zip › File S1.pdf]

SERVIZIO SANITARIO REGIONALE
EMILIA-ROMAGNA
Azienda Unità Sanitaria Locale di Reggio Emilia
IRCCS Istituto in tecnologie avanzate e modelli assistenziali in oncologia

OECI CLINICAL Cancer Centre

Fatigue, cachessia e attività fisica

Come affrontare stanchezza e perdita di peso

Testi a cura di

Monia Allisen Accogli, fisioterapista, Medicina Fisica e Riabilitativa, Arcispedale Santa Maria Nuova, Azienda USL - IRCCS di Reggio Emilia.

Loredana Buonaccorso, psicologa psicoterapeuta, Unità di Psico-oncologia, Arcispedale Santa Maria Nuova, Azienda USL - IRCCS di Reggio Emilia.

Monica Denti, fisioterapista, Medicina Fisica e Riabilitativa, Arcispedale Santa Maria Nuova, Azienda USL - IRCCS di Reggio Emilia.

Stefania Fugazzaro, medico fisiatra, Medicina Fisica e Riabilitativa, Arcispedale Santa Maria Nuova, Azienda USL - IRCCS di Reggio Emilia.

Silvia Tanzi, medico palliativista, Unità di Cure Palliative, Arcispedale Santa Maria Nuova, Azienda USL - IRCCS di Reggio Emilia.

Illustrazioni a cura di

Carlotta Mainini, fisioterapista, Medicina Fisica e Riabilitativa, Arcispedale Santa Maria Nuova, Azienda USL - IRCCS di Reggio Emilia.

Revisione linguistica e testuale

Elena Cervi, infermiera, In-Forma Salute, Biblioteca Medica, Arcispedale Santa Maria Nuova, Azienda USL - IRCCS di Reggio Emilia.

Simone Cocchi, bibliotecario, Biblioteca Medica, Arcispedale Santa Maria Nuova, Azienda USL - IRCCS di Reggio Emilia.

Ultimo aggiornamento: **giugno 2020**

Indice

Introduzione 4
La fatigue e la cachessia 4
  Cosa sono 4
  L'impatto sulle emozioni e sulla famiglia 5
  Il trattamento della cachessia e dei disturbi causati dalla cachessia 6
Pianifica le attività 8
  Come posso pianificare le attività? 8
  E se non riesco a raggiungere l'obiettivo? 8
Fai attività fisica 9
  Quali attività posso fare? 9
  Può essere pericoloso fare attività fisica? 9
  Come posso pianificare l'attività fisica? 10
  Come posso monitorare la mia attività ? 11
Esercizi di rinforzo muscolare 12
  Arti inferiori 12
  Tronco 15
  Arti superiori 16
Esercizi di rinforzo con elastici 16
Esercizi per la flessibilità/stretching 18
  Arti inferiori 18
  Collo e schiena 20
  Arti superiori 21
La respirazione diaframmatica: rilassarsi respirando 22
Contatti 23
Bibliografia 24
Allegati 25
1. Il mio piano di attività 26
2. Il mio piano di attività 28
3. Il mio piano di attività 30

Introduzione

Questo opuscolo è stato creato per le persone con diagnosi di cachessia in carico presso l'Unità di Cure Palliative che partecipano al progetto "**Intervento psico-educativo e riabilitativo per il paziente oncologico con cachessia e il suo caregiver: uno studio di fattibilità**". Il progetto si propone di verificare se un programma psico-educativo associato ad un programma riabilitativo incentrato sull'attività fisica può aiutare a gestire meglio la cachessia e a ridurre la fatigue.
In questo opuscolo troverai le informazioni e i punti chiave che verranno affrontati negli **incontri di psico-educazione** (con le infermiere) e negli **incontri riabilitativi** (con le fisioterapiste) previsti dal progetto. Le informazioni sono pensate per esserti di supporto durante il programma e di aiuto in qualsiasi altro momento del percorso di cura. Non è detto che le informazioni fornite qui e durante gli incontri seguano lo stesso ordine.
Le informazioni contenute in questo opuscolo non intendono sostituire il colloquio diretto con i professionisti sanitari. Usa questo opuscolo come spunto per domande e richieste.

La fatigue e la cachessia

Cosa sono

La stanchezza (detta anche astenia) è uno dei sintomi principali delle persone con un tumore, sia che facciano chemioterapia o immunoterapia sia che non la facciano. In particolare, la cosiddetta **fatigue** è una stanchezza associata al tumore che viene definita come "una sensazione soggettiva, stressante, persistente di stanchezza o spossatezza correlata al cancro o al suo trattamento non proporzionale all'attività eseguita, che interferisce con le abituali attività e che spesso non è alleviata dal sonno o dal riposo"[1].
Questo significa che una persona può provare concretamente questo tipo di stanchezza anche se non ha fatto nulla di particolarmente faticoso. Il tumore infatti, provoca uno stato di infiammazione cronica e, a causa di questo, è come se l'organismo fosse sempre sotto sforzo. Questo stato di infiammazione cronica causa, quindi, la fatigue e una situazione clinica definita **cachessia** (o,

[1] (National Comprehensive Cancer Network –NCCN Guidelines).

più precisamente, *sindrome anoressia-cachessia* o *malnutrizione associata al tumore*).

Una persona con cachessia può:

- mangiare poco;
- non avere affatto appetito;
- sentirsi subito sazia, anche se ha mangiato molto poco;
- sentire dei sapori diversi nel cibo;
- perdere peso;
- riuscire a fare solo poca attività fisica o non riuscire affatto a farne;
- perdere massa muscolare, anche molta ad esempio nelle braccia, nelle gambe, nel volto, ecc.;
- fare fatica a concentrarsi;
- perdere interesse per le attività quotidiane;
- sentirsi senza forze e senza energie sia dal punto di vista fisico che psichico.

L'impatto sulle emozioni e sulla famiglia

Il cibo non è importante solo perché serve per nutrire il corpo. Il cibo ha anche un valore psicologico, emotivo, sociale e culturale. Spesso veniamo educati a dare valori e significati particolari al cibo. Alcune persone, ad esempio, credono che chi ha un buon appetito sia sano e cresca forte e che chi si sforza di mangiare migliorerà il suo appetito. Sono di uso comune frasi come *"se mangia vuol dire che sta bene", "se non mangia c'è qualcosa che non va"*; *"l'appetito vien mangiando", "se si sforza di mangiare, starà meglio"* e così via.
Altre persone hanno imparato che cucinare per il proprio caro è un gesto di affetto e di grande cura, perché cucinare significa anche nutrire e aiutare la persona a stare meglio. Anche da un punto di vista sociale il cibo accompagna ricorrenze particolari (Natale, Pasqua, compleanni, ecc.) o semplicemente è un momento per stare con la famiglia o con gli amici. In generale, quindi, attraverso il cibo si possono trasmettere dei veri e propri gesti cura.
Una persona che perde involontariamente peso a causa della cachessia vede cambiare il suo aspetto fisico e questo può portare a sentirsi demoralizzati, a volte a provare ansia e a evitare le relazioni con i familiari e gli amici. Spesso tutto questo è dovuto a un senso di vergogna per il cambiamento dell'immagine di sé o all'imbarazzo nel non riuscire più a mangiare come prima. La persona non si riconosce più, fa fatica ad accettare l'involontario dimagrimento, di conseguenza perde interesse per il cibo e il suo appetito cala ancora di più.

Sempre a causa della cachessia ci possono essere cambiamenti importanti nelle abitudini alimentari che modificano l'equilibrio nella relazione di coppia o, in generale, in famiglia. I pasti diventano situazioni difficili e non più momenti per stare bene insieme.
I familiari vogliono aiutare e si impegnano a cucinare i cibi che più piacciono al proprio caro, dedicano tempo a fare la spesa, a scegliere con cura la qualità dei cibi e anche a trovare ricette che possano stimolare l'appetito. Tutto questo può portare ad avere comportamenti di forzatura all'alimentazione. **La cachessia, però, non si risolve solo mangiando di più.**
Può anche succedere di conseguenza che la persona malata si chiuda ancora di più in sé stessa, per evitare conflitti con i familiari che impiegano tempo e cura nella preparazione dei cibi. I famigliari a loro volta percepiscono, nel rifiuto del loro cibo cucinato con cura, un mancato riconoscimento del loro impegno e del loro ruolo nell'assistenza. Questo può causare sensi di colpa e uno stato di ansia.

Parlare di questa condizione, di cosa la causa e di come affrontarla **è quindi essenziale**. Nella nostra esperienza, per gestire gli effetti della cachessia è importante imparare a **rapportarsi col cibo in modo diverso**, provare a **condividere un po' di più le proprie emozioni** e trovare modi per **prendersi cura del proprio caro non solo attraverso il cibo**.

La gestione di questa sindrome **non si può improvvisare** perché provare a mangiare di più e cibi diversi non è sufficiente. Affrontare la cachessia richiede uno spazio dedicato nel quale **poter parlare di come ci si sente** rispetto al cibo, **confrontandosi** anche **con il proprio familiare** più vicino e **con gli operatori** che sono esperti nella gestione di questa sindrome.

L'Unità di Cure Palliative e l'Unita di Psico-Oncologia sono a disposizione tua e dei tuoi cari per approfondire questi argomenti e per aiutarti ad affrontare la situazione col giusto approccio, per poter migliorare la qualità di vita tua e della tua famiglia.

Il trattamento della cachessia e dei disturbi causati dalla cachessia

Per affrontare la fatigue e la cachessia è necessario agire su più fronti. Per questo motivo, quindi, il trattamento deve essere gestito da diversi esperti.

Questo è quello che può fare il **medico**:

- correggere l'anemia (bassi valori di emoglobina, disturbi degli elettroliti e altri valori del sangue come il sodio, il potassio, il magnesio e quelli relativi al funzionamento della tiroide);
- trattare i sintomi (nausea, dolore, difficoltà a respirare, depressione, ansia);
- considerare i possibili effetti collaterali di alcuni farmaci (farmaci contro il dolore, chemioterapia, ecc…);
- trattare i disturbi del sonno (a questo proposito si rimanda all'opuscolo 'Consigli per un buon sonno');
- usare farmaci, come psicostimolanti o cortisone per brevi periodi (altri farmaci, come i supporti di vitamine, si sono dimostrati inutili).

Questo, invece, è quello che puoi fare **tu**:

- mangia più cibi ricchi di calorie e proteine, nel rispetto di quello che riesci a fare.
- Cerca di ridurre lo stress. In generale, se puoi, cerca di evitare discussioni o conflitti con i tuoi cari. Prova a concentrare le energie sul tuo stato psico-fisico. Condividi quello che provi o quello che vorresti fare. Cerca anche di ascoltare diversi punti di vista o consigli, ma senza forzare cambiamenti se non ti senti di farlo. Presta attenzione alle tue emozioni ed evita di concentrarti solo sul cibo.
- Gestire al meglio l'energia che hai a disposizione (a questo proposito ti consigliamo di leggere l'opuscolo *Suggerimenti per risparmiare energia*). Un modo efficace per gestire le energie è quello di pianificare le proprie attività.
- Fai più attività fisica. Quest'ultima sembra essere la cosa più importante soprattutto per i pazienti in chemioterapia.

Dato che per pianificare le attività e fare attività fisica sono due aspetti molto importanti, nella restante parte dell'opuscolo trovi utili indicazioni ed esempi per poter raggiungere questi obiettivi.

Per ogni necessità, contatta l'**Unità di Cure Palliative** al numero **0522 295329** (dal **lunedì** al **venerdì**, ore **9-10** e **12-13.30**).

Pianifica le attività

Un modo per contrastare la fatigue è pianificare le attività della giornata**.** Quando pianifichi le attività, infatti, riesci a organizzarti in modo da gestire al meglio le tue energie.

Come posso pianificare le attività?

Puoi mettere in atto alcune strategie del cosiddetto "lavoro per obiettivi". Le persone che definiscono degli obiettivi, infatti, realizzano molte più cose nell'arco della giornata.
Un obiettivo è una tappa intermedia nel percorso. Per arrivare a destinazione, le tappe intermedie devono essere realistiche e significative.
Stabilire obiettivi può aiutarti anche ad acquisire una maggiore consapevolezza dei tuoi limiti e delle tue capacità.

Gli obiettivi possono essere:

- **a breve termine,** se li puoi raggiungere in una o due settimane;
- **a lungo termine,** se li puoi raggiungere in qualche settimana o mese.

Se l'obiettivo è troppo grande da raggiungere tutto in una volta, puoi suddividerlo in "passi" più piccoli o in compiti più "realizzabili".

E se non riesco a raggiungere l'obiettivo?

Puoi procedere così:

- **identifica il problema** che ti ha impedito di raggiungere l'obiettivo;
- **fai una lista di idee** per risolvere il problema;
- **scegli un'idea** da provare;
- **valuta i risultati:** se il problema è risolto, bene! Altrimenti…
- **…prova una delle altre idee della lista** e continua a valutare i risultati, finché la lista è esaurita. Se non riesci a risolvere il problema…
- **…impiega altre risorse:** chiedi altre idee ad amici, familiari od operatori sanitari. Scegli una di queste idee e ripeti il procedimento. Se il problema non è ancora risolto, allora…
- **…accetta il fatto che forse non puoi risolvere ora il problema.** Puoi provare a risolverlo in un altro momento, ad esempio dopo una settimana di ulteriore attività fisica.

Fai attività fisica

Fare attività fisica è uno degli strumenti principali per gestire la fatigue legata alla cachessia. Muoversi, infatti, aiuta a muoversi sempre di più. Inoltre, fare attività fisica aiuta a:

- stimolare l'appetito
- sentirsi meglio
- ridurre altri fattori che peggiorano la cachessia.

Quali attività posso fare?

In questo momento del tuo percorso, alcuni tipi di attività fisica sono più consigliati di altri per gestire la fatigue legata alla cachessia. Ognuna di queste attività ha scopi ed effetti differenti.

FORZA	ELASTICITÀ	RILASSAMENTO
A corpo libero<br>Con pesi<br>Con elastici	Stretching<br>Yoga<br>Tai Chi e Qi Gong	Respirazione diaframmatica<br>Attività di immaginazione guidata

Può essere pericoloso fare attività fisica?

Fare attività è generalmente sicuro, segui però questi consigli. In ogni caso, se hai dubbi rivolgiti al tuo medico di riferimento.

Cosa fare
Usa calzature adatte (ad es. con suola in gomma antiscivolo).
Bevi almeno 6-8 bicchieri d'acqua al giorno (circa 1 litro e mezzo). Questo ti aiuterà a prevenire la disidratazione.
Dopo l'attività, fai una merenda "sana" con frutta o verdura (e non con dolci o cibi confezionati).

Interrompi sempre l’attività se:
hai dolore al petto;
fai fatica a respirare;
hai un mal di testa che non passa;
ti gira la testa;
ti senti debole;
le ossa o la schiena ti fanno male in modo diverso dal solito.

NON fare attività fisica se:
non ti senti bene;
hai la febbre o un’infezione in atto.

Come posso pianificare l’attività fisica?

Ti proponiamo di usare il **piano di attività**. Si tratta di una tabella che ti aiuta a programmare l’attività da svolgere a casa.
Per compilarlo devi dare una risposta a queste domande:

- **Che cosa?** (quale esercizio tra quelli proposti dal fisioterapista vuoi fare)
- **Ripetizioni?** (quante ripetizioni per ogni esercizio pensi di fare)
- **Quali problemi potresti incontrare?** (quali pensi possano essere gli ostacoli che potrebbero impedirti di raggiungere il tuo obiettivo?)
- **Livello di convinzione pari a 7 o più** (in una scala che va da 0 a 10, dove 0=nessuna convinzione e 10=assoluta convinzione, quanto sei sicuro/a di riuscire a completare questa attività? Se la risposta è inferiore a 7, prova a modificare il piano di attività per essere più convinto/a di riuscire. Avere successo è importante!)

Nella sezione **Allegati** troverai gli schemi da compilare che ti aiuteranno a pianificare al meglio le attività.

Come posso monitorare la mia attività ?

È importante che tu tenga traccia dell'attività fisica che svolgi in autonomia a casa attraverso un **diario di attività**. Nella sezione **Allegati** troverai uno schema che ti aiuterà a farlo.

Per ogni tipo di attività, nelle prossime pagine trovi indicati diversi esercizi che puoi fare e quante volte a settimana ti consigliamo di farli.

Esercizi di rinforzo muscolare

2
volte/sett

Con questi esercizi i muscoli lavorano in modo intenso e diventano quindi più forti. Anche l'equilibrio e la coordinazione migliorano. Questi esercizi possono essere fatti senza resistenza oppure con pesi o elastici (contro resistenza).
La scelta degli esercizi deve essere personalizzata. Se hai dei dubbi, chiedi al tuo medico e/o al fisioterapista.

Arti inferiori

1. Tricipite surale (polpaccio)

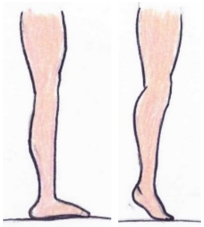

a. Spinte in piedi
Appoggiati ad un tavolo o al muro, alzati sulle punte con entrambi i piedi e scendi lentamente. Ripeti 20 volte.

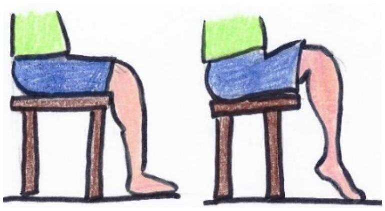

b. Spinte da seduto
Da seduto/a, spingi sulle punte dei piedi, staccando i talloni da terra, e scendi lentamente.
Ripeti 20 volte.

c. Spinte con elastico
Sdraiati a pancia in su con una gamba dritta e l'altra piegata, metti l'elastico sotto la pianta del piede della gamba distesa e tiralo. Spingi verso il basso la punta del piede e ritorna lentamente alla posizione iniziale.
Ripeti 20 volte.

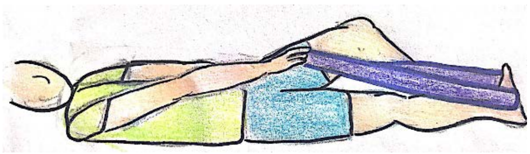

2. Quadricipite (Coscia anteriore)

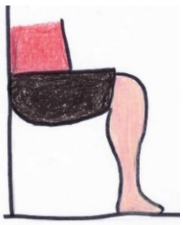

a. Squat al muro/alzata dalla sedia
Appoggiati al muro con la schiena e piegati sulle ginocchia. Rimani nella posizione per almeno 10 secondi e rialzati lentamente.
Puoi anche stringere un cuscino fra le ginocchia, per aumentare la contrazione dei muscoli della coscia.

b. Da seduto/a

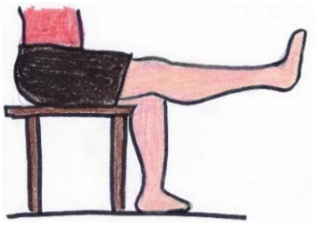

Stendi un ginocchio per volta e mantieni la posizione per almeno 5 secondi. Ripeti 10 volte per gamba.
Puoi anche stringere un cuscino fra le ginocchia, per aumentare la contrazione dei muscoli della coscia.

c. Sdraiato/a a pancia in su

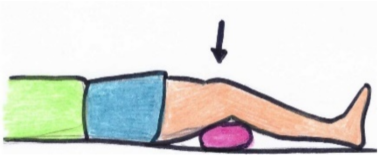

Metti un cuscino sotto al ginocchio, schiaccialo e tieni la posizione per 5 secondi. Rilascia e ripeti 10 volte per gamba.

3. Estensori d'anca (Coscia posteriore)

a. Da sdraiato/a a pancia in giù

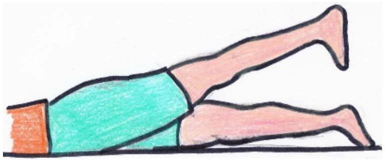

Porta in alto un piede, mantenendo il ginocchio disteso, contraendo i glutei (senza sforzare la schiena). Scendi lentamente.
Ripeti 10 volte per gamba.

b. Ponte

Sdraiati a pancia in su, appoggia i piedi a terra aperti alla larghezza del bacino, tieni le braccia lungo il corpo e i palmi delle mani appoggiati a terra. Mentre inspiri solleva il bacino e contrai i glutei (senza usare la schiena). Mentre espiri, scendi lentamente e torna alla posizione iniziale. Ripeti 10 volte.

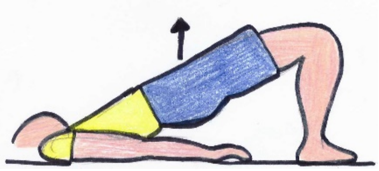

c. Gatto con estensione

Appoggiati con mani e ginocchia a terra. Tieni dritta la schiena e solleva una gamba estesa. Oppure solleva la gamba con il ginocchio piegato per usare maggiormente i glutei. Riappoggia il ginocchio e ripeti 10 volte per gamba.

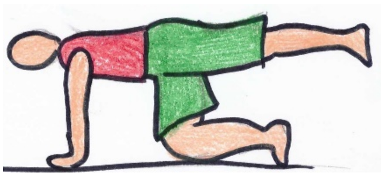

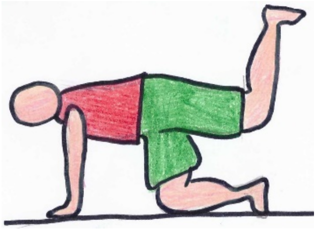

d. In piedi appoggiato/a al muro o al tavolo

Tieni la gamba dritta e porta indietro il piede, senza spostarti avanti con la schiena.
Ritorna alla posizione di partenza e ripeti 10 volte per gamba.

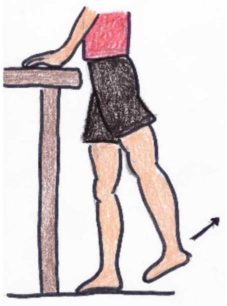

4. Adduttori (interno coscia)

a. Da sdraiato/a a pancia in su o seduto/a

Tieni le ginocchia piegate e un cuscino tra le gambe
Schiaccia il cuscino tra le gambe e mantieni la posizione per 5 secondi. Rilascia, senza far cadere il cuscino e ripeti 15 volte.

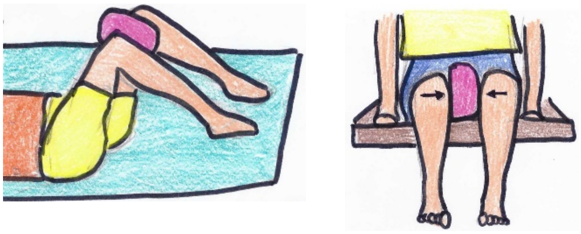

b. In piedi con elastico

Metti l'elastico a livello della caviglia. Tieni la gamba distesa e avvicina il piede all'altra gamba, in chiusura. Rilascia lentamente e ripeti 10 volte per gamba.

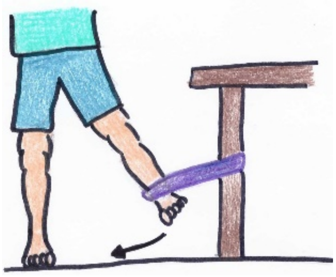

5. Abduttori (esterno coscia)

a. Da sdraiato/a a pancia in su o seduto/a

Metti l'elastico intorno alle ginocchia piegate.
Apri le ginocchia in fuori, poi rilascia lentamente. Ripeti 15 volte.

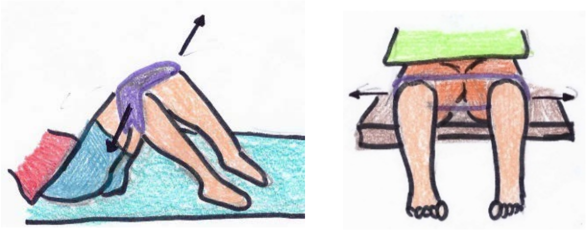

b. In piedi con elastico

Metti l'elastico intorno alle caviglie. Appoggiati di lato al muro o ad un tavolo, apri la gamba esterna, mantenendo il ginocchio esteso. Ritorna lentamente. Ripeti 10 volte per gamba.

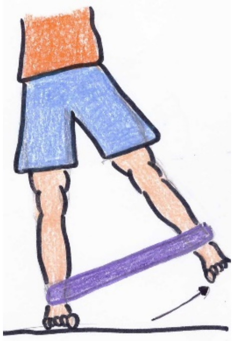

Tronco

6. Addominali

Da sdraiato/a a pancia in su

Tieni le ginocchia piegate e le braccia distese lungo il corpo.
Mentre **espiri** contrai l'addome, solleva la testa da terra e porta le braccia verso i talloni. Mentre **inspiri** ritorna lentamente alla posizione di partenza. Ripeti 10 volte.

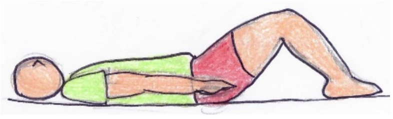

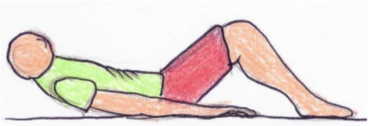

7. Erettori spinali

a. **Da sdraiato/a a pancia in su o seduto/a**

Avvicina le scapole, portando indietro i gomiti, mantenendo le spalle basse. Mantieni la posizione 5 secondi e rilascia. Ripeti 10 volte.

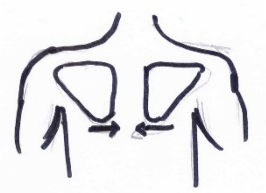

b. **Da sdraiato/a a pancia in giù**

Braccia lungo il corpo, fronte appoggiata a terra.
Solleva le spalle, le braccia e la testa e, mentre continui a guardare a terra, porta le mani verso i piedi. Mantieni la posizione 5 secondi e ritorna lentamente. Ripeti 10 volte.

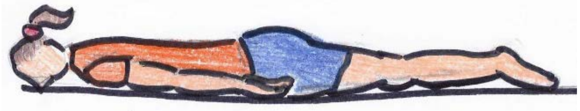

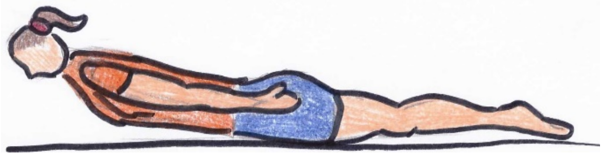

Arti superiori

8. Esercizi a corpo libero o con pesi

a. **Da sdraiato/a a pancia in su o seduto/a**
Unisci le mani e tieni i gomiti stesi.
Porta le braccia verso l'alto, il più possibile sopra la testa. Non portare le spalle verso le orecchie, ma cerca di tenerle basse.
Ritorna alla posizione di partenza. Ripeti 10 volte.

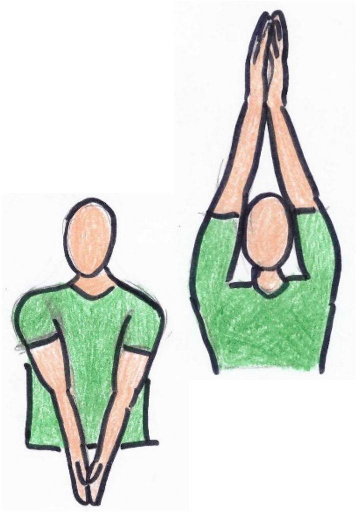

b. **Da seduto/a o in piedi**
Braccia lungo i fianchi, tieni i gomiti dritti e apri le braccia lateralmente, portandole in alto il più possibile. Ritorna alla posizione di partenza, controllando di non alzare le spalle.
Ripeti 10 volte.

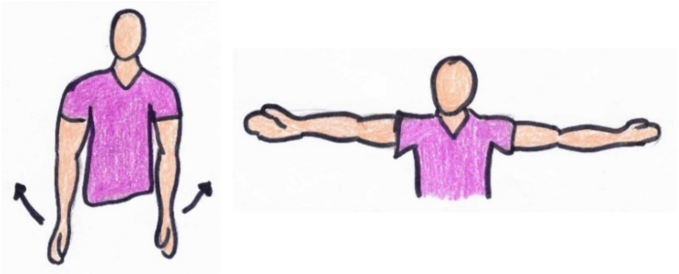

N.B.: puoi eseguire questi esercizi usando un peso da mezzo kilo (0,5 Kg) o una bottiglietta d'acqua da mezzo litro (0,5 L).

Esercizi di rinforzo con elastici

Nel fare questi esercizi, parti con l'elastico tirato al minimo e allungalo.

9. Stabilizzatori di scapola

In piedi
Fissa l'elastico alla maniglia della porta. Parti con il braccio steso davanti a te. Porta il braccio indietro e verso il basso tirando l'elastico fino al bacino.
Ritorna lentamente alla posizione iniziale. Ripeti 10 volte

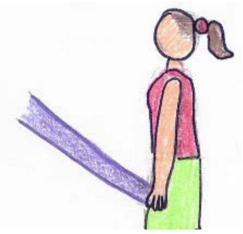

10. Tricipite brachiale

In piedi

Fissa l'elastico alla gamba del tavolo. Appoggia il petto al tavolo lasciando fuori la spalla e il braccio. Tira l'elastico indietro, mantenendo il gomito disteso.
Ritorna lentamente. Ripeti 10 volte

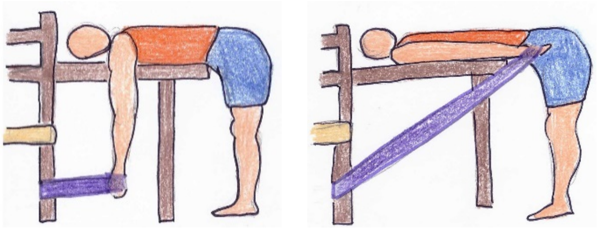

11. Rotatori esterni

In piedi o da seduto/a

Braccia attaccate al corpo, e gomiti piegati a 90°. Con un braccio tira l'elastico verso l'esterno, mantenendo il gomito vicino al corpo.
Ritorna alla posizione di partenza lentamente.
Ripeti 10 volte per braccio.

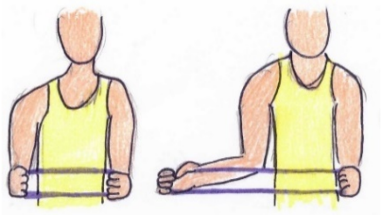

12. Rotatori interni

In piedi o da seduto/a

Fissa l'elastico alla maniglia di una porta, parti con il braccio attaccato al corpo, gomito piegato a 90°. Tira l'elastico verso la pancia, mantenendo il gomito vicino al corpo. Ritorna alla posizione di partenza lentamente.
Ripeti 10 volte per braccio.

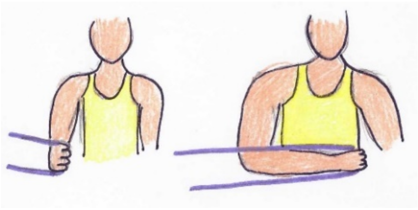

13. Adduttori

In piedi

Fissa l'elastico alla maniglia della porta.
Parti con il braccio staccato dal corpo, lateralmente. Mantenendo il gomito esteso avvicina il braccio al corpo e ritorna lentamente. Ripeti 10 volte.

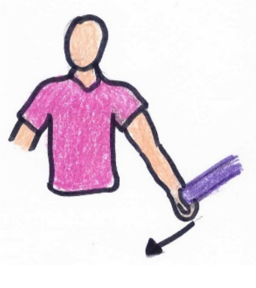

Esercizi per la flessibilità/stretching

2
volte/sett

Allungano o sciolgono i muscoli e le articolazioni ed aiutano l'equilibrio e la coordinazione.

Bisogna eseguirli lentamente, mantenendo la posizione per almeno **20 secondi**. In questo modo i tuoi muscoli avranno la possibilità di rilassarsi e allungarsi.
Puoi fare questi esercizi in qualsiasi momento della giornata; sono però più efficaci "a caldo", cioè dopo aver fatto attività fisica, quando i muscoli sono più plastici.

Arti inferiori

14. Tricipite surale (polpaccio)

In piedi
Appoggia i gomiti al muro e metti un piede avanti all'altro. Piega il ginocchio della gamba davanti mentre tieni i talloni bene appoggiati a terra.
Ripeti con l'altra gamba.

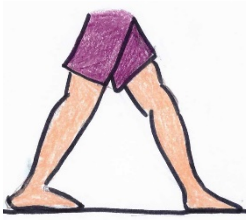

15. Quadricipite (coscia anteriore)

In piedi appoggiato/a al muro o sdraiato sul fianco
Afferra la caviglia e piega il ginocchio per allungare i muscoli anteriori della coscia. Ripeti con l'altra gamba.

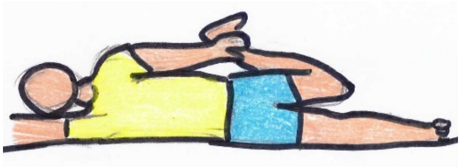

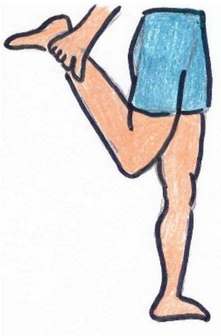

16. Glutei/piriforme

Sdraiato/a a pancia in su

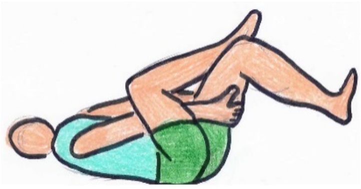

Appoggia la caviglia sul ginocchio opposto, afferra la gamba sotto con entrambe le mani e avvicina le gambe al petto. Se non riesci puoi appoggiare il piede al muro. Sentirai tirare il gluteo e i muscoli posteriori della coscia della gamba accavallata.
Ripeti con l'altra gamba.

17. Muscoli posteriori della coscia

Sdraiato/a a terra con le gambe appoggiate al muro

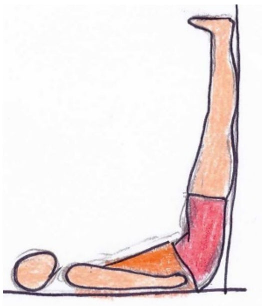

Meno tensione senti dietro alle ginocchia e più puoi avvicinare il sedere al muro. Per maggior comodità puoi mettere un cuscino sotto la testa.
Mantieni la posizione con le ginocchia il più possibile distese, respira con la pancia per almeno 1 minuto.
Ti consigliamo di incrementare gradualmente il tempo in base alla tua tolleranza.

18. Ileo-Psoas

Sdraiato/a a pancia in su

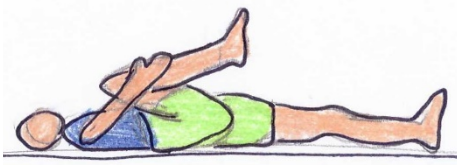

Porta e mantieni un ginocchio al petto. Sentirai tensione nella parte anteriore dell'anca della gamba distesa.
Ripeti con l'altra gamba.

19. Adduttori (interno coscia)

In piedi

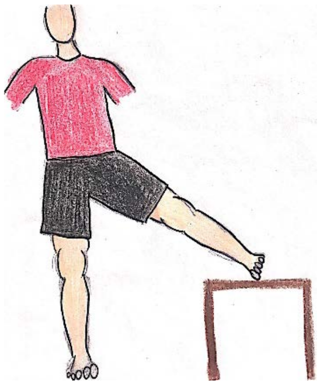

Appoggia una gamba su un piano rialzato (es. sedia), tieni la punta del piede rivolta in avanti e il ginocchio esteso. Sentirai tirare i muscoli dell'interno coscia. Per maggiore sicurezza puoi appoggiarti con le mani per esempio al tavolo.

Collo e schiena

20. Collo

a. Inclina la testa di lato (orecchio verso la spalla) per allungare i muscoli laterali del collo;
b. piega in avanti la testa, portando il mento verso la gola, per allungare i muscoli posteriori del collo e del dorso.

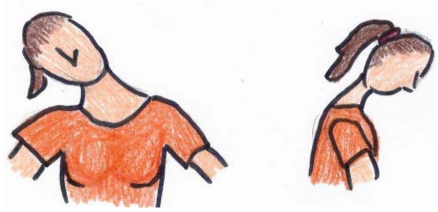

21. Schiena

a. Sdraiato/a a pancia in su

porta le ginocchia al petto (entrambe o una alla volta) e mantieni la posizione. Per maggior comodità puoi mettere un cuscino sotto la testa.

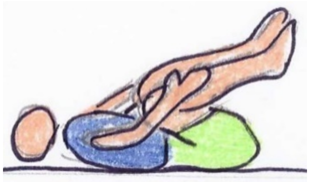

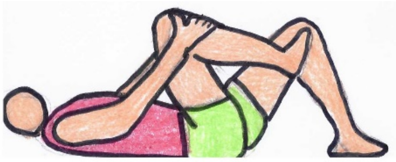

b. In ginocchio

Scivola in avanti con le braccia e indietro con il sedere, portando il petto verso il pavimento. Sentirai tensione alle braccia e al dorso.
Mantieni la posizione e respira profondamente con la pancia.

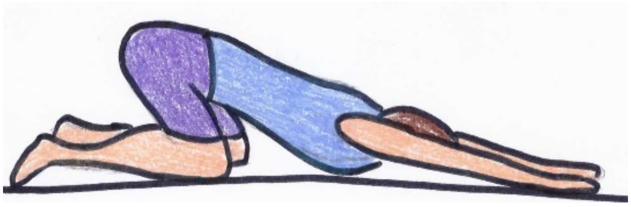

Arti superiori

22. Tricipite

In piedi o da seduto/a

Porta la mano dietro alla schiena passando da sopra la testa oppure porta il braccio davanti al collo col gomito piegato.

Sentirai tensione nella parte posteriore della spalla e del braccio.

Ripeti con l'altro braccio.

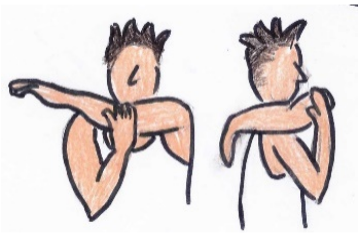

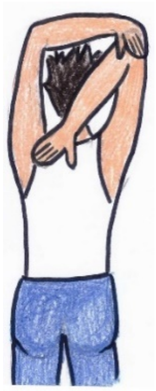

23. Pettorale (al muro, 3 posizioni per i 3 fasci)

In piedi

Appoggia mano, gomito e spalla al muro; lentamente gira lo sguardo, il petto e il bacino dalla parte opposta, per mettere in tensione la parte superiore del petto.

Puoi mettere il braccio come in figura a 3 altezze diverse, per uno stretching più completo dei tre diversi fasci del muscolo pettorale.

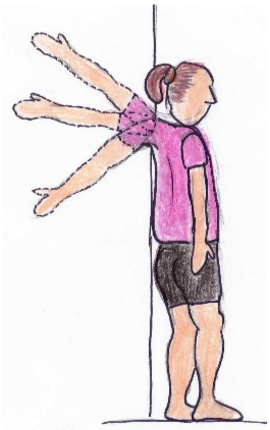

24. Flessori del polso

In piedi o da seduto/a

Stendi il gomito con il palmo della mano verso il basso. Aiutati con l'altra mano e tira le dita verso l'alto, verso di te. Mantieni la posizione e ripeti con l'altro braccio.

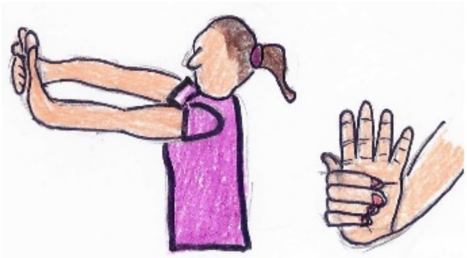

25. Estensori del polso

In piedi o da seduto/a

Stendi il gomito con il palmo della mano verso il basso. Aiutati con l'altra mano e tira le dita verso il basso. Mantieni la posizione e ripeti con l'altro braccio.

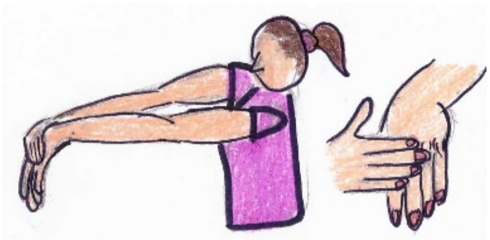

La respirazione diaframmatica: rilassarsi respirando

La respirazione è un'attività fondamentale della nostra vita, di cui non ci accorgiamo. Respirare bene non solo dà benessere in condizioni normali, ma può essere un utile strumento per gestire alcuni sintomi.
Per respirare bene è necessario usare nel modo giusto uno dei muscoli coinvolti nella respirazione, cioè il diaframma.

La respirazione diaframmatica può essere utile per:

- liberare la tensione e rilassarsi;
- recuperare quando manca il respiro, sia a causa di una malattia cronica, sia per una malattia acuta come il raffreddore;
- recuperare da uno sforzo quando si fa attività fisica.

La respirazione è composta da 2 fasi, entrambe molto importanti:

a. **inspirazione**: dal naso si riempiono i polmoni di aria, ricca di ossigeno;

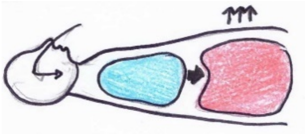

b. **espirazione**: dalla bocca si svuotano i polmoni ed espelliamo anidride carbonica. Questa fase dovrebbe durare circa il doppio dell'inspirazione.

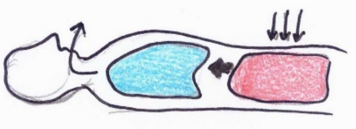

Per una corretta respirazione diaframmatica, fai così:

- Sdraiati con le ginocchia piegate e rilassati (si può fare anche da seduti prestando attenzione a rilassare le spalle).

- Metti una mano sulla pancia appena sopra l'ombelico.

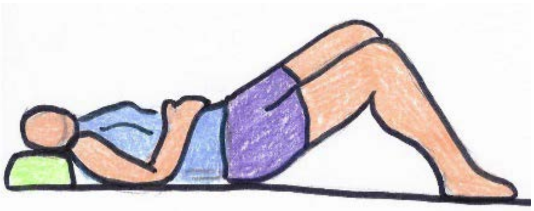

- Inspira dal naso, se riesci, altrimenti inspira dalla bocca. Dovresti sentire l'addome che si solleva.

- Poi espira lentamente attraverso le labbra socchiuse. Lascia che l'aria fuoriesca lentamente, **NON** forzarla.

Le persone che si affaticano rapidamente o con problemi di respirazione cercano spesso di affrettare le attività, per finire prima di restare senza fiato.
Se anche tu tendi a fare così, rallenta e usa moderazione. Usa questa tecnica di respiro per 3-4 minuti, e alternala con l'attività che ti sei proposto di fare. In questo modo, ti sentirai più attivo durante la giornata.

Contatti

Unità di Cure Palliative

Tel. **0522 295329**

dal **lunedì** al **venerdì**

dalle ore **9** alle ore **10** e dalle ore **12** alle ore **13.30**.

Bibliografia

Mc Millan Cancer Support, www.macmillan.org.uk

National Comprehensive Cancer Network –NCCN Guidelines, www.nccn.org

K.H. **Schmitz** et al. 2010, American College of sports medicine roundtable on exercises guidilines for cancer survivors Medicine & Science in Sports & Exercise. 42(7):1409-1426, JUL 2010

Self-Management Resource Center www.selfmanagementresource.com

Allegati

Data __/__/____

1. Il mio piano di attività

In queste due settimane io voglio fare…

Esercizio	Ripetizioni	Quali problemi potrei incontrare?

Quanto sei convinto da 0 a 10? __/10

Diario di attività

Giorno	Esercizi	Fatica
o 1 _________ __/__/____		___/10
o 2 _________ __/__/____		___/10
o 3 _________ __/__/____		___/10
o 4_________ __/__/____		___/10
o 5_________ __/__/____		___/10
o 6_________ __/__/____		___/10

Note:____

Data __/__/____

2. Il mio piano di attività

In queste due settimane io voglio fare…

Esercizio	Ripetizioni	Quali problemi potrei incontrare?

Quanto sei convinto da 0 a 10? __/10

Diario di attività

Giorno	Esercizi	Fatica
o 1 ________ __/__/____		___/10
o 2 ________ __/__/____		___/10
o 3 ________ __/__/____		___/10
o 4________ __/__/____		___/10
o 5________ __/__/____		___/10
o 6________ __/__/____		___/10

Note:____

Data __/__/____

3. Il mio piano di attività

In queste quattro settimane io voglio fare…

Esercizio	Ripetizioni	Quali problemi potrei incontrare?

Quanto sei convinto da 0 a 10? __/10

Diario di attività

Giorno	Esercizi	Fatica
o 1 _________ ___/___/		___/10
o 2 _________ ___/___/		___/10
o 3 _________ ___/___/		___/10
o 4 _________ ___/___/		___/10
o 5 _________ ___/___/		___/10
o 6 _________ ___/___/		___/10

Note:____

Diario di attività

Giorno	Esercizi	Fatica
o 7 _________ ___/___/		___/10
o 8 _________ ___/___/		___/10
o 9 _________ ___/___/		___/10
o 10 _________ ___/___/		___/10
o 11 _________ ___/___/		___/10
o 12 _________ ___/___/		___/10

Note:____
