# Supplementary material for: Psycho-Educational and Rehabilitative Intervention to Manage Cancer Cachexia (PRICC) for Advanced Patients and Their Caregivers: Lessons Learned from a Single-Arm Feasibility Trial
Source: Cancers (Basel). 2023 Mar 30;15(7):2063. doi: 10.3390/cancers15072063 (PMC10093308; doi:10.3390/cancers15072063)
Supplement: Supplementary file 1 [file cancers-15-02063-s001.zip › File S2.pdf]

File S2: QUESTIONS FOR DYADS AND HEALTHCARE PROFESSIONALS

STUDY: Psycho-educational and rehabilitative intervention to manage cancer cachexia (PRICC) for patients and their caregivers

1. Questions addressed to the patient-caregiver dyad:

a) Can you tell us how it went when you were asked to participate in the research project?

b) How was the project explained to you? From who?

c) Have you had the opportunity to talk about this proposal together? Have you talked to anyone about it?

d) What did you think about participation/non-participation? What thoughts have you had? How did you feel?

Questions related to the psycho-educational intervention:

a) Can you tell me how the meetings with the nurses went? What did she offer you?

b) What favoured the path that has been proposed to you? (Investigate in particular the times, methods, and places, if they were adequate, if they were supportive)

c) What hindered the path that was proposed to you?

d) Do you have any suggestions to help us improve this intervention?

Questions related to the rehabilitation intervention:

a) Can you tell me how the physiotherapy sessions in the hospital went? What did they offer you?

b) What did they tell you?

c) Can you tell me how the physiotherapy sessions at home went?

In your experience, did the two interventions integrate well into the path that was offered to you?

2. Questions addressed to nurses and physiotherapists who participated in the study

a) Can you tell us how your participation in the study went?

b) What favoured your intervention?

c) What hindered your intervention?

d) Do you have any suggestions to help us improve this intervention?

In your experience, have the two interventions integrated well into the pathway offered to patients and family members?
